# Nanostructured Formulations for a Local Treatment of Cancer: A Mini Review About Challenges and Possibilities

**DOI:** 10.3390/pharmaceutics17020205

**Published:** 2025-02-06

**Authors:** Tatiane Roquete Amparo, Tamires Cunha Almeida, Lucas Resende Dutra Sousa, Viviane Flores Xavier, Glenda Nicioli da Silva, Geraldo Célio Brandão, Orlando David Henrique dos Santos

**Affiliations:** 1 Department of Pharmacy, Federal University of Ouro Preto, Rua Professor Paulo Magalhães Gomes, 122-Bauxita, Ouro Preto 35400-000, Brazil; lucasresendedutrasousa@gmail.com (L.R.D.S.); viviane.flores.xavier@gmail.com (V.F.X.); nicioli@ufop.edu.br (G.N.d.S.); celiobrandao@ufop.edu.br (G.C.B.); orlando@ufop.edu.br (O.D.H.d.S.); 2 Laboratory of Pain and Signaling, Butantan Institute, Av. Vital Brasil, 1500–Butantã, São Paulo 05503-900, Brazil; tamires-cunha@hotmail.com

**Keywords:** nanoformulations, nanotechnology, cancer, local treatment

## Abstract

Cancer represents a significant societal, public health, and economic challenge. Conventional chemotherapy is based on systemic administration; however, it has current limitations, including poor bioavailability, high-dose requirements, adverse side effects, low therapeutic indices, and the development of multiple drug resistance. These factors underscore the need for innovative strategies to enhance drug delivery directly to tumours. However, local treatment also presents significant challenges, including the penetration of the drug through endothelial layers, tissue density in the tumour microenvironment, tumour interstitial fluid pressure, physiological conditions within the tumour, and permanence at the site of action. Nanotechnology represents a promising alternative for addressing these challenges. This narrative review elucidates the potential of nanostructured formulations for local cancer treatment, providing illustrative examples and an analysis of the advantages and challenges associated with this approach. Among the nanoformulations developed for the local treatment of breast, bladder, colorectal, oral, and melanoma cancer, polymeric nanoparticles, liposomes, lipid nanoparticles, and nanohydrogels have demonstrated particular efficacy. These systems permit mucoadhesion and enhanced tissue penetration, thereby increasing the drug concentration at the tumour site (bioavailability) and consequently improving anti-tumour efficacy and potentially reducing adverse effects. In addition to studies indicating chemotherapy, nanocarriers can be used as a theranostic approach and in combination with irradiation methods.

## 1. Introduction

Cancer is a disease characterized by the uncontrolled proliferation of transformed cells [1]. These cells are subject to evolutionary selection, including genetic and epigenetic changes, and are capable of spreading to other parts of the body [1]. The pathophysiology of cancer encompasses a multitude of intricate hallmarks, including the capacity to sustain proliferative signalling, evade growth suppressors, resist cell death, achieve replicative immortality, induce angiogenesis, activate invasion and metastasis, and ultimately, genome instability [2].

Cancer represents a significant societal, public health, and economic challenge in the 21st century [3]. Updated estimates from the International Agency for Research on Cancer (IARC) indicate that there were nearly 20 million new cases of cancer in 2022. These diseases are among the leading causes of mortality globally, with an estimated one in nine men and one in twelve women dying from them [3]. The high mortality and morbidity rates are indicative of the complexity of the disease and the limitations of available treatment modalities, which encompass surgical, chemotherapeutic, radiotherapeutic, and immunotherapeutic approaches [4]. While traditional treatments may potentially delay the progression of cancer, they frequently entail significant adverse effects and may be constrained in their efficacy, particularly in advanced disease stages [5].

It is therefore imperative that new treatments need to be developed, and nanotechnology represents an interesting alternative through which efficacy can be enhanced while adverse effects can be reduced. Nanomedicines have the potential to enhance drug solubility and stability, which may result in increased bioavailability and more efficacious outcomes for patients [6]. Furthermore, functionalization enables the targeted delivery of drugs to specific areas, thereby reducing the adverse effects associated with damage to healthy cells, which represents a significant challenge in cancer treatment [7].

Several studies have demonstrated the benefits of local treatment for various types of cancer [8,9,10,11,12,13]. However, the scope of these studies is limited to a single type of cancer or a specific nanostructure. Additionally, some these studies focus on immunotherapy, while others do not address the application of nanotechnology. Therefore, the objective of this narrative review is to highlight the potential of nanostructured formulations for local cancer treatment, including bladder, breast, colon, lung, oral, and skin cancers. Illustrative examples are provided, and the advantages and challenges associated with this approach are discussed.

## 2. Advantages and Challenges of the Local Treatment of Cancer

The conventional chemotherapy is accomplished via systemic administration, whereby the majority of antineoplastic drugs are delivered via intravenous or oral routes [14]. This form of delivery is widely used, and advances in systemic therapy, both in the adjuvant and metastatic settings, have resulted in increased cure rates and overall survival [15]. It offers several advantages, including predictable serum pharmacokinetics and the simplicity and pervasiveness of the required infrastructure. Consequently, it is the conventional approach employed in drug development [15].

However, systemic treatment has several disadvantages, including low bioavailability at the tumour site, which is often due to the tumour’s dense stroma and abnormal vasculature, which obstruct drug penetration [16]. To circumvent this obstacle, higher doses are necessary, which in turn gives rise to elevated toxicity in normal cells and an increased incidence of multiple drug resistance [17]. The severe adverse effects induced by chemotherapeutic drugs on healthy tissues and organs, despite causing unintended and undesirable side effects, e.g., the loss of appetite and nausea, are a significant factor contributing to the high mortality rate of cancer patients [17].

It is therefore evident that the current limitations of systemic chemotherapy, including poor bioavailability, high-dose requirements, adverse side effects, low therapeutic indices, and the development of multiple drug resistance, underscore the need for innovative strategies to enhance drug delivery directly to tumours [18,19,20]. There are two principal methods of locoregional delivery: direct infusion, which encompasses intratumoural injections or implanted ports that permit direct drug access to a tumour site; and drug-loaded depots, which include implants that can be placed in or near the tumour/resection cavity or in situ-forming drug reservoirs that are injected in/near the tumour/resection site [13].

Localized cancer treatments may result in greater drug concentration at the site of action, thereby improving therapeutic outcomes while minimizing systemic toxicity [9,11,12]. It seems probable that local delivery will play a significant role in achieving long-term cancer-free survival in patients. Furthermore, it may be combined with other available systemic therapies and physical local strategies, such as radiotherapy. This represents a promising field for the next generation of cancer treatments, offering the potential for continued improvement in cancer therapy without additional toxicity [13].

**Figure 1:**
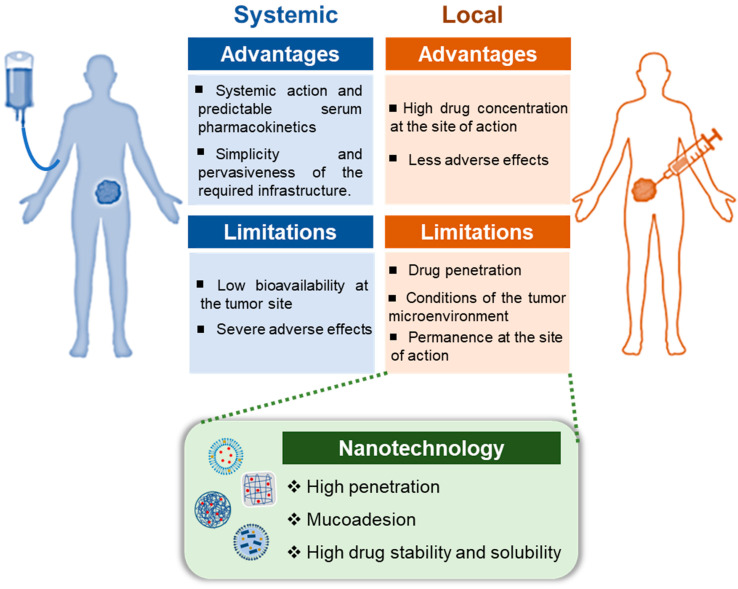
Advantages and limitations of systemic and local administration of anticancer agents and the potential of nanotechnology to address the challenges of local treatment.

However, these therapies also present significant challenges such as the penetration of the drug through the endothelial layers, tissue density in the tumour microenvironment, tumour interstitial fluid pressure, physiological conditions within the tumour, and permanence at the site of action (due to the effects of secretions/physiological fluids and local pH) [9,11,13]. Nanotechnology represents a promising approach for addressing these challenges (Figure 1).

## 3. Nanocarrier Drug Delivery Systems

Nanocarriers are defined as colloidal particles ranging in size of 1000 nm or less [21]. These formulations can be administered in the body via various routes, including intravenous, intraperitoneal, transdermal, oral, ocular, intranasal, and intratumoural. The choice of the system type and its physical characteristics (size, shape, viscosity, and material) depends on the target and the route of delivery [9].

Nanoparticles for drug delivery can be composed of a variety of materials and exhibit diverse structural characteristics. These include organic materials such as polymers [22], liposomes [23], dendrimers [24], and nanoemulsions [25], as well as inorganic nanoparticles comprising magnetic, gold, or silica [26]. Additionally, quantum dots and carbon-based materials (nanotubes) are also utilized in this field [27].

**Figure 2:**
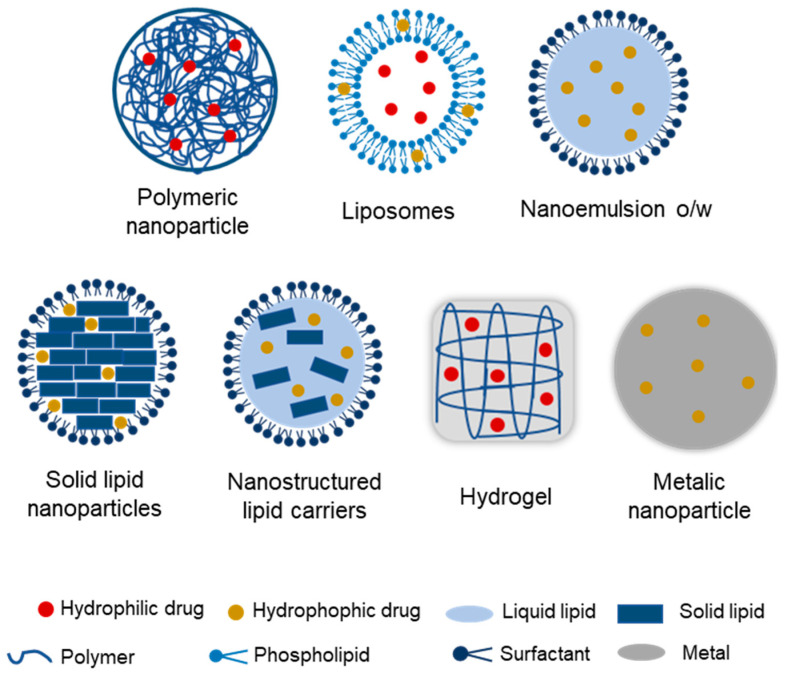
Nanostructured systems frequently employed for local drug delivery in cancer treatment.

Organic nanocarriers demonstrate an enhanced capacity to load drugs and exhibit higher biocompatibility [27]. Among the organic nanocarriers most commonly employed for localized delivery in cancer therapy are polymeric nanoparticles, liposomes, nanoemulsions, solid lipid nanoparticles, nanostructured lipid carriers, and hydrogels (Figure 2).

Polymeric nanoparticles are systems in which the active compound is entrapped within or adsorbed onto the polymeric core (natural or synthetic), comprising nanocapsules and nanospheres, respectively [28]. Liposomes, nanoemulsions, solid lipid nanoparticles, and nanostructured lipid carriers are examples of lipid-based nanocarriers. Liposomes are spherical lipid vesicles composed of one or more lipid bilayers with a hydrophilic core [29]. Nanoemulsions are defined as fine water-in-oil (w/o) and oil-in-water (o/w) dispersions of two immiscible fluids [25]. Solid lipid nanoparticles are lipid emulsions in which the liquid lipid (oil) has been substituted by a solid lipid [21]. Nanostructured lipid carriers in turn contain a lipid mixture consisting of both solid and liquid lipids in their core [30]. Nanocomposite hydrogels are defined as hybrid materials comprising three-dimensional polymer networks and additional elements that confer enhanced elasticity and strength [31]. 

Inorganic nanocarriers are also a possibility to increase the effectiveness and lessen the adverse effects of drugs [27]. These systems are nanospheres that consist of a core region comprising an inorganic constituent (silica, silver, gold, or iron oxide covered with organic polymers, which offers an appropriate substrate to conjugate biomacromolecules or guard the core from undesirable physical and chemical interactions with the exterior biologic microenvironment [27]). Among these, the metallic nanoparticles are the most commonly used for the local treatment of cancer (Figure 2).

## 4. Nanoformulations for the Local Treatment of Cancer

Although local cancer therapy has a number of advantages, it is not a viable option for all types of cancer. In this review, we focused on bladder, breast, colorectal, lung, melanoma, and oral cancers, which are among the most prevalent cancer types and have been the subject of the most extensive research in this area.

The local administration of bladder cancer treatment can be performed via intravesical drug delivery, which is the direct instillation of medicinal drugs into the bladder through a catheter. This approach offers significant advantages over conventional drug delivery systems for drug administration, as it enhances efficacy while circumventing systemic off-target side effects [32]. Nevertheless, this method continues to exhibit limitations associated with the physiology of the urinary bladder. These limitations include the difficulty of maintaining optimal drug concentrations due to regular voiding and limited penetration that result from the poor permeability of the urothelium [33]. The aforementioned challenges can be overcome by encapsulating drugs in nanoformulations [11,33].

The treatment of solid tumours, such as melanoma, breast, and oral cancer, may be achieved through intratumoural administration via injection or implants. When administered in this manner, the drugs initially disperse throughout the injection site, thereby creating a high local tissue concentration. Over time, they gradually enter the systemic circulation, allowing for a controlled absorption process. This pharmacokinetic behaviour can enable the use of higher doses with improved tolerability [12].

Oral cancer and melanoma can also be locally treated via the topical administration of the drug, as the nanocarriers are an important approach to improve retention at the application site (mucoadhesion) and the penetration into the epithelium [8,34].

In the context of colorectal cancer, the oral colon-targeted drug delivery system comprises a drug delivery strategy designed to deliver therapeutic agents to the site of colonic disease. Oral nanocarriers modified with different materials facilitate the penetration of the drug through the intestinal mucus barrier while simultaneously protecting it from the acidic environment of the gastrointestinal tract, which represents significant challenges to local delivery to colorectal tumours [10].

Local treatment also has advantages for lung cancer, since the delivery of systemic drugs is limited due to the difficulty of passage through the blood–air barrier. The bioavailability of nanoparticles administered via inhalation depends on the size, shape, and surface of the nanoparticles, which define their superior aerodynamics, allowing for higher retention and low drug loss following delivery [9].

The following sections present examples of nanoformulations developed for the local treatment of these cancers.

### 4.1. Bladder Cancer

Bladder cancer is the most common malignancy of the urinary tract and is highly prevalent worldwide [35]. This type of cancer is generally categorized as either non-muscle-invasive or muscle-invasive, based on the tumour’s spread and the depth of the invasion into the bladder wall [36]. Bladder cancer has the highest lifetime treatment costs per patient among all cancers, largely due to its high recurrence rate and the need for ongoing invasive monitoring [37].

The treatment of bladder cancer depends on the disease stage and tumour characteristics and may include surgery, localized or systemic chemotherapy, and radiation therapy [36]. For instance, in cases of non-muscle-invasive bladder cancer, intravesical chemotherapy is recommended following transurethral resection to reduce the risk of recurrence [38]. However, a key challenge remains, namely the limited residence time of the drugs within the bladder. Several potential options for addressing this issue are provided below (Table 1).

Guo et al. (2020) [40] developed a positively charged, disulfide-crosslinked nanogel composed of oligoarginine-poly(ethylene glycol)-poly(L-phenylalanine-co-L-cystine) to improve the mucoadhesiveness and penetrability of chemotherapeutic drugs. The study used 10-hydroxycamptothecin as a model drug. In vitro experiments demonstrated that the formulation enhanced cellular uptake and increased the cytotoxicity. In a rat model of bladder cancer, the fluorescence intensity of the nanogel formulation decreased over time, but it remained at a relatively high concentration in the bladder even 12 h after administration, indicating strong mucoadhesion to the urothelial surface. In contrast, the fluorescence signal of free 10-hydroxycamptothecin rapidly decreased, attributed to continuous urine excretion. The formulation also showed enhanced penetration into the bladder, with drug accumulation primarily in the bladder, 2.2 times higher than free drug, and minimal distribution in other organs. Regarding the efficacy, the nanogel significantly reduced tumour growth, which led to a stable increase in body weight and improved survival rates in both rats and mice.

In the study by Wang et al. (2020) [41], researchers explored the use of poly(amidoamine)-modified mesoporous silica nanoparticles as a drug delivery system to doxorubicin. Poly(amidoamine) was highlighted for its innate bioadhesive properties, while silica provided an ideal structure with interstitial spaces for loading and transporting drugs to tumour sites. The study revealed that doxorubicin-loaded nanoparticles exhibited reduced cytotoxicity in UM-UC-3 bladder cancer cells compared to free doxorubicin, which was attributed to the gradual release of the drug within the cells. Moreover, the nanoparticles demonstrated significantly higher mucoadhesivity on the bladder wall compared to free doxorubicin. This enhanced mucoadhesive property, combined with controlled drug release, is expected to prolong drug residence time on the bladder wall, offering potential benefits for intravesical chemotherapy.

Xu et al. 2020 [39] developed a glutathione (GSH)-responsive drug delivery system based on positively charged chitosan, a well-established mucoadhesive biomolecule. This system was designed to enhance drug contact with the bladder, improving tissue permeability and residence time. Firstly, researchers synthesized a reactive oxygen species (ROS)-responsive prodrug of gambogic acid, enabling the selective delivery of the active drug to cancer cells, since they presented higher intracellular ROS levels compared to normal cells. They also demonstrated that the prodrug exhibited improved release in a medium containing high ROS and GSH levels, enhancing drug selectivity. Compared to the GSH-responsive system, the non-responsive formulation showed lower cytotoxicity and reduced effectiveness. In vivo studies confirmed that the system increased mucoadhesiveness and enhanced bladder penetration relative to the free drug. Both responsive and non-responsive formulations inhibited tumour growth effectively. Importantly, the treatment exhibited high selectivity, with no evidence of urothelial damage, weight loss, and toxicity in major organs such as the heart, lungs, kidneys, liver, or spleen.

**Figure 3:**
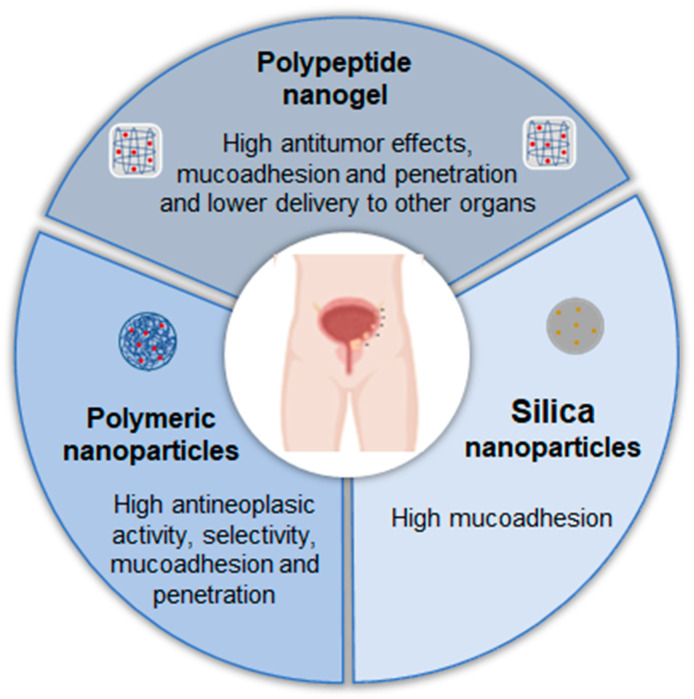
Examples of nanostructured systems already studied and their advantages for the local treatment of bladder cancer.

In summary, nanostructured formulations represent a promising path for the intravesical treatment of bladder cancer, offering several advantages in efficacy (Figure 3).

### 4.2. Breast Cancer

Breast cancer is the most commonly diagnosed cancer in women and the leading cause of cancer-related deaths in this population [35]. Notably, it is estimated that in 2020, around one million children became maternal orphans due to their mothers dying from cancer. Nearly half of these cases were attributed to maternal deaths from either breast or cervical cancer [42].

Breast cancer is classified into immunohistochemical subtypes based on the presence or absence of estrogen, progesterone, and human epidermal growth factor receptors [43]. Given its heterogeneity, management strategies must consider the specific subtype of the disease. Recently, innovative drug delivery strategies, such as nanostructured systems, have shown promise for enhancing local treatment. These approaches include intraductal administration or targeting drugs to the breast, offering potential benefits such as prolonged tissue retention, improved targeting and selectivity, increased cytotoxicity, and reduced administration frequency [44]. In this context, some progress has already been made to improve the local treatment of breast cancer (Table 2).

In a study against breast cancer, An et al. (2020) [48] developed Cu^2^⁺-doped zeolitic imidazolate frameworks coated with polydopamine nanoparticles (PDA@Cu/ZIF-8 NPs) for glutathione (GSH)-triggered and photothermal-enhanced sequential catalytic therapy. These nanoparticles were engineered to exploit the elevated GSH levels in cancer cells for targeted therapeutic effects. The PDA@Cu/ZIF-8 NPs reacted with intracellular GSH to deplete GSH while producing Cu⁺ ions, which facilitated hydroxyl radical (·OH) generation. This process enabled controlled reactive oxygen species (ROS) production and GSH depletion specifically at tumour sites. Additionally, the polydopamine (PDA) shell, a highly effective photothermal agent in the near-infrared (NIR) region, generated substantial heat under 808 nm laser irradiation [49]. This heat amplified both ROS generation and GSH depletion, further inhibiting tumour growth. The nanoparticles demonstrated negligible cytotoxicity in normal cells due to the low GSH and H₂O₂ levels in healthy cells. Conversely, the viabilities of tumour cell lines (MCF-7, A549, and MDA-MB-231) decreased in a dose-dependent manner when exposed to PDA@Cu/ZIF-8 NPs. In vivo studies on mice with breast cancer confirmed the efficacy of the nanoparticles. The intratumoural injection of PDA@Cu/ZIF-8 NPs significantly reduced tumour growth, with even greater reductions observed when combined with laser treatment. Importantly, no toxicity was detected in major organs (heart, liver, spleen, lungs, and kidneys), underscoring the safety and biocompatibility of the nanoparticle system.

In the study carried out by Oltolina et al. (2020) [46], magnetic nanoparticles mediated by magnetosome proteins for the delivery of doxorubicin were designed to enhance drug delivery to tumour sites using an external magnetic field. The application of a magnetic field significantly increased the cellular uptake of doxorubicin-loaded nanoparticles compared to the treatment without a magnetic field or with free doxorubicin. In vivo studies demonstrated a pronounced reduction in tumour growth in animals treated with nanoparticles of doxorubicin under the influence of a magnetic field. Notably, all treatments, including nanoparticles without a magnetic field, free doxorubicin, and nanoparticles without doxorubicin, showed initial anti-tumour effects, particularly during the early observation period. The enhanced performance of nanoparticles under a magnetic field was attributed to their ability to concentrate at the tumour site, maximizing localized drug effects while minimizing systemic side effects in other organs. Moreover, the combination of doxorubicin nanoparticles with an alternating magnetic field induced localized hyperthermia (~43 °C), a temperature at which tumour cells are particularly vulnerable [50]. This hyperthermic effect selectively targeted tumour cells without damaging healthy tissue, further enhancing therapeutic outcomes.

Al-Zubaydi et al. (2022) [47] developed a nanoscale drug delivery system for the localized treatment of ductal carcinoma in situ, a noninvasive breast cancer characterized by the proliferation of cancerous epithelial cells within the mammary ducts [51]. Due to limited blood circulation to the mammary ducts, systemic drug administration often results in subtherapeutic drug concentrations, underscoring the need for targeted local treatments [52]. The researchers designed an esterase-responsive prodrug of ciclopirox to exploit the high esterase activity in tumour tissues. Two formulations were evaluated: polymeric nanoparticles with sustained drug release properties and a nanosuspension of nanocrystals stabilized with an amphiphilic polymer, which allowed for high drug loading and immediate release. In vivo studies demonstrated that the nanosuspension of the ciclopirox prodrug significantly enhanced drug persistence in mammary tissue compared to both free ciclopirox and the nanosuspension containing unmodified CPX. Regarding anti-tumour efficacy, the combination of the prodrug nanosuspension with prodrug-loaded nanoparticles yielded superior results, showcasing sustained and localized drug delivery.

Li et al. (2024) [45] explored the use of trypsin to improve the tumour distribution of liposomes loaded with gambogic acid. This innovative approach addresses a critical challenge in nanomedicine, which is the dense tumour stroma and tight cellular junctions that hinder the penetration of therapeutic agents into tumours [53]. The rationale for using trypsin lies in its ability to degrade the dense stroma, which consists of specialized connective tissues and the extracellular matrix. These structures act as physical barriers, reducing the efficacy of nanomedicines and contributing to the failure of several formulations in clinical trials [54]. Previous in vitro studies demonstrated that trypsin enhances the penetration of nanoformulations by digesting membrane proteins on tumour cells and disrupting tight junctions between endothelial cells. In vivo, the combination of gambogic acid liposomes with a trypsin–hydrogel treatment significantly reduced tumour weight in tumour-bearing mice.

**Figure 4:**
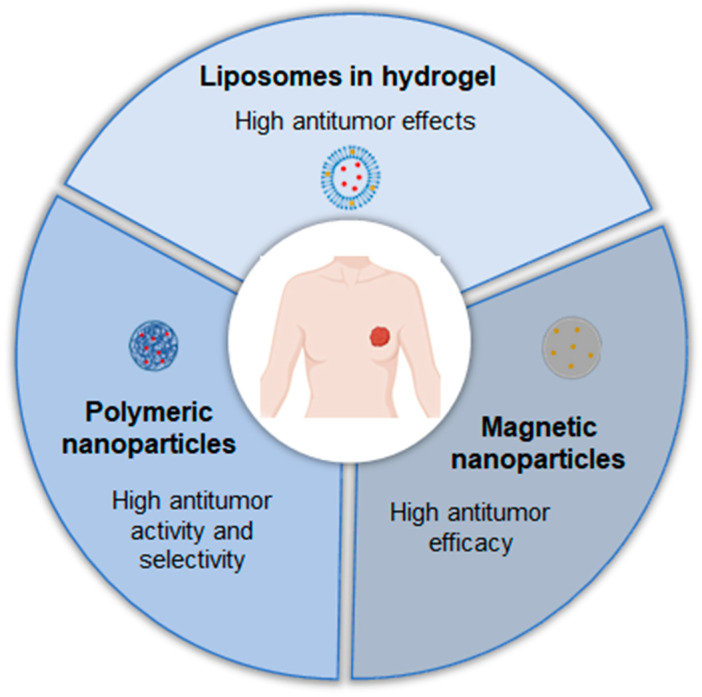
Examples of nanostructured systems already studied and their advantages for the local treatment of breast cancer.

In summary, nanostructured formulations could be an improved tool for the intravesical treatment of breast cancer, offering several advantages in efficacy (Figure 4).

### 4.3. Colorectal Cancer

Colorectal cancer is one of the most prevalent forms of cancer globally, ranking as the third most commonly diagnosed cancer in 2020 [55]. Moreover, its prevalence is higher in young adults, which further exacerbates the concern. The prevalence of this disease varies considerably between different geographical regions, with higher rates observed in developed countries. This can be attributed to a number of factors, including alcohol consumption, tobacco use, and a sedentary lifestyle [55,56].

Colorectal cancer is frequently classified based on the site of origin: colon cancer, rectal cancer, and rectosigmoid junction cancer [57,58]. The majority of cases are adenocarcinomas, which originate from the glandular cells of the intestinal epithelium [59]. In an attempt to address these forms of colorectal cancer, nanostructured formulations are being developed for local treatment (Table 3).

Polymeric nanoparticles are the most commonly used nanocarriers for the local treatment of colorectal cancer. Tian and Wu et al. (2023) [62] developed a novel nanocomposite comprising bufadienolide molecules decorated with a chitosan quaternary salt for targeted release in the colonic environment. The formulation exhibited enhanced solubility and stability of bufadienolides, in addition to promoting controlled and targeted release at acidic pH. Another advantage was the increased residence time in the colon, which facilitated localized drug release and enhanced the bioavailability of the active ingredient. In vitro and in vivo studies demonstrated high cytotoxicity, the induction of apoptosis, and increased reactive oxygen species (ROS) against tumour cells, which resulted in a reduction in tumour growth, confirming the efficacy of the formulation.

In a related development, Chen, Zhao, and Xu et al. (2020) [68] created functionalized chitosan nanoparticles for photodynamic and photothermal therapies against colorectal cancer. The rationale for this approach was that the association could enhance anti-tumour effects. While photodynamic therapy would promote the production of ROS, photothermal therapy would increase the local temperature, thereby sensitizing cells and intensifying cytotoxicity. The results demonstrated enhanced cytotoxicity and the cellular internalization of nanoparticles, which were accompanied by elevated tumour temperature and oxidative stress, as indicated by elevated levels of reactive oxygen species, superoxide, and singlet oxygen. Following oral administration in vivo, there was increased biodistribution, a reduction in tumour growth and volume, and a favourable safety profile.

Zhu et al. (2023) [66] developed dual-targeted colonic nanoparticles for oral administration. These were magnetically actuated and loaded with chlorogenic acid using pectin and iron oxide modified with oleic acid. The proposed therapy combines the bioactivity of chlorogenic acid with the magnetic targeting of the nanoparticles to optimize delivery and therapeutic effects in the colon. This resulted in a significant increase in cellular internalization and cytotoxicity, with evidence of induction of apoptosis in tumour cells.

Abbasi et al. (2023) [70] devised an oral delivery system comprising a hydrogel containing folic acid-functionalized alginate nanoparticles that encapsulate diferoylmethane. The nanoparticles were functionalized with folic acid, thereby enhancing their targeting of tumour cells that overexpress folate receptors. Indeed, an increase in the internalization of the nanoparticles was observed, which may be related to the increased cytotoxicity observed in cancer cells. Furthermore, in the in vivo administration model, a three-fold increase in the peak maximum concentration and an extension of the mean residence time were observed.

In addition to polymeric nanoparticles, other systems have also been developed. Lei et al. (2020) [61] developed an immunogenic therapy system that employs a protamine-liposome complex to facilitate the delivery of the cytokine IL-15 in conjunction with transcribed mRNA. IL-15 was selected for its anti-tumour activity, which stimulates the immune system. The system was efficacious in increasing cancer cell apoptosis and reducing the number of nodules, tumour weight, and growth, in addition to decreasing angiogenesis and metastasis. The formulation demonstrated potential for the efficient targeting of the tumour microenvironment, local and systemic immune stimulation with minimal toxicity, and as a possible combination therapy. The local and systemic administration of the complex exhibited an inhibition greater than 50% in the C26 abdominal cavity metastasis tumour model and in the subcutaneous and lung metastasis model, indicating high efficacy and safety. In a subsequent approach, Zahiri and Babaei (2020) [69] developed dendritic mesoporous silica nanoparticles to carry doxorubicin. The formulation was designed to selectively target cancer stem cells by combining an RNA aptamer against CD133 with dextran polycarboxylic acid, and it demonstrated efficacy in increasing cytotoxicity and cellular internalization.

In order to load a chemotherapeutic agent and a photothermal agent, Su et al. (2024) [63] created nanodiamonds coated with polydopamine modified with amino-functionalized polyethylene glycol and triphenylphosphine coated with Eudragit S100 containing doxorubicin. The polydopamine present in the formulation demonstrated efficacy in generating heat to kill cancer cells, which contributed synergistically with the chemotherapeutic agent doxorubicin. Furthermore, the formulated product, when administered orally, was observed to enhance the efficacy of the active agents by increasing their absorption by cancer cells and prolonging their residence time in the colon (due to the presence of triphenylphosphine) while simultaneously protecting them from degradation. It is plausible that these properties were responsible for enhancing the cytotoxicity and apoptosis of tumour cells, which would also be associated with the reduction in tumour weight and growth.

Additionally, Wang and Zhang et al. (2022) [60] developed stable, modified mesoporous carbon nanoparticles containing folic acid and the M27-39 peptide for oral administration, with the objective of targeting colon cancer mitochondria. The findings revealed enhanced cellular internalization of the formulation, accompanied by an elevated generation of reactive oxygen species. This may have resulted in irreversible mitochondrial damage, ultimately leading to high levels of cytotoxicity and the induction of apoptosis in tumour cells. Furthermore, the nanostructured system exhibited augmented accumulation in the colon and extended residence time at the site, which may have contributed to the reduction in tumour growth and the number of tumours per animal.

Additionally, Rashidzadeh et al. (2023) [64] developed a pH- and enzyme-responsive polymeric nanohydrogel containing methotrexate and chloroquine for the treatment of colorectal cancer. The formulation exhibited controlled and targeted release in response to characteristics of the tumour microenvironment, such as acidic pH and enzymes, thereby optimizing local delivery. In vitro studies indicated high cytotoxicity, while in vivo assays suggested that the developed nanocarrier is safe. In a similar context, Lu et al. (2022) [71] developed solid lipid nanoparticles in a microgel containing cisplatin and SPIONs for the oral administration of a chemo/magnetothermal combination against colon cancer. The findings demonstrated augmented cellular internalization and cytotoxicity, in addition to enhanced accumulation within the colon. The formulation demonstrated efficacy in reducing tumour growth and metastasis while exhibiting minimal toxicity to healthy tissues. In a related study, Abdullah et al. (2021) [65] developed an oral nanosuspension containing 5-fluorouracil, an anticancer drug with a short biological half-life. The coated particles were encapsulated in an aqueous hydrodynamic gel comprising sodium alginate and an excess of lambda-carrageenan or chitosan. In vitro assays utilizing HCT-116 cells demonstrated augmented apoptosis and caspase-9 activation, which are associated with the elevated cytotoxicity also observed.

Zhao and Du et al. (2020) [67] developed a novel approach using peptosomes co-encapsulated in oxidized glucomannan-based microspheres to simultaneously transport curcumin and a microRNA-31 inhibitory oligonucleotide. One of the advantages of this system is its mucoadhesive characteristic, which would be beneficial for the treatment of colorectal cancer. In vitro and in vivo models demonstrated that the treatment suppressed tumour growth through increased cellular internalization, cytotoxicity, and a reduction in tumour growth and volume, as well as the number of tumours per mouse. These effects can be attributed to an increase in mucoadhesion, the retention of active ingredients, and local bioavailability.

**Figure 5:**
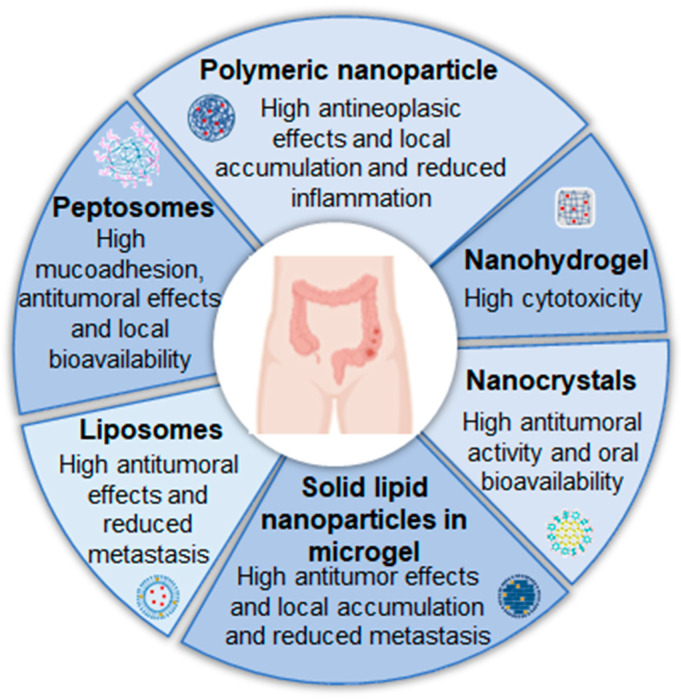
Examples of nanostructured systems already studied and their advantages for the local treatment of colorectal cancer.

In summary, nanostructured formulations showed several benefits when applied for the intravesical treatment of colorectal cancer, offering several advantages in efficacy (Figure 5).

### 4.4. Lung Cancer

According to the International Agency for Research on Cancer (IARC), lung cancer is the neoplasm with the highest morbidity and mortality in the world. The latest global estimate made in 2022 showed that this type of cancer is responsible for almost one in eight (12.4%) cancers diagnosed and one in five (18.7%) cancer deaths [3]. Lung cancer is classified according to its histological characteristics and is divided into two large groups: small cell carcinomas and non-small cell carcinomas [72]. This second group, in addition to being the most common, affecting around 80% of patients, can be divided into three main subtypes: adenocarcinoma, squamous cell carcinoma, and large cell carcinoma [72,73].

Different therapeutic approaches have been developed to fight lung cancer. The choice of treatment depends directly on the stage of the disease, the patient’s clinical condition, the regions affected, and the tolerance to medication. Often, in the early stages, surgery is a potentially curative option. However, as the disease progresses to more advanced stages, other therapeutic modalities are needed, such as chemotherapy, radiotherapy, immunotherapy, targeted therapy, or a combination of treatments [74].

Even with a range of treatment options, lung cancer is among the neoplasms with the lowest cure rate, mainly due to the difficulty in establishing an early diagnosis and resistance to therapy [75]. For this reason, the different scenarios that make up the disease must be analyzed at all times so that it is possible to propose approaches that can meet all the challenges of clinical practice [76]. Thus, new systems for administering chemotherapy agents directly into the lungs have been developed by various research groups (Table 4).

The most cited type of nanocarrier in the studies evaluated was the liposome. Studies carried out in vitro and ex vivo showed that liposomes loaded with pirfenidone promoted a 1.5- to 2-fold reduction in colony growth compared to the treatment with plain pirfenidone and also led to a significant increase in cytotoxicity and apoptosis and a decrease in cell migration, angiogenesis, and metastasis [78]. The liposome’s encapsulation of paclitaxel enhanced the expressions of immune markers (TNF-α, IL-4, and IFN-γ) and immune cells (leukocytes and neutrophils) and increased bioavailability and cytotoxicity, evaluated in vitro and in vivo [79].

Sarvepalli et al., 2022 [80] verified through in vitro tests that the encapsulation of indomethacin in the liposome led to a significant increase in bioavailability, cytotoxicity, and caspase 3 activation, as well as a decrease in cell colony growth, tumour progression and COX-2. Sawant et al., 2021 [81] also found that the active and passive liposomes of osimertinib reduced IC_50_ by 2.2 and 1.2 times, respectively, compared to the free drug; the authors also found that there was an increase in its bioavailability and a decrease in cell migration, cell colony growth, and tumour size; these results were obtained through in vitro tests.

Some authors have studied nanoparticle systems, such as Ma et al., 2022 [83] who encapsulated the K4 monomer in this system and verified that there was an increase in cytotoxicity and mucus penetration; results were verified through in vitro and in vivo tests. Satari et al., 2020 [84] encapsulated gefitinib in nanoparticles and found through in vitro tests that the nanoformulation developed had a superior anticancer effect compared to free gefitinib, and increased cytotoxicity and cellular internalization were observed. Ray et al., 2024 [85] developed a nanoparticle containing niclosamide, and through in vitro studies, they found that there was a significant increase in cytotoxicity, apoptosis, cellular internalization, and autophagolysosomes and a decrease in cell colonies and alveolar dysplasia. Pre-clinical trials have shown considerable tumour regression with minimal adverse effects.

Other systems have also been described in the literature such as cubosomes, which are lipid vesicles comparable to vesicular systems like liposomes but characterized as cubic-shaped lyotropic liquid crystalline nanoparticles [86]. Patil and colleagues, 2021 [77] developed cubosomes and encapsulated bedaquiline; in vitro tests showed that there was a significant increase in cytotoxicity, apoptosis, and bioavailability and a decrease in cell migration, metastasis, and tumour growth. In addition, Chauhan et al., 2023 [82] prepared nanoemulsions of the tyrosine kinase inhibitor erlotinib. This system showed a twofold lower IC_50_ for the erlotinib-loaded nanoemulsion compared to the erlotinib-free solution and promoted a significant decrease in tumour volume.

**Figure 6:**
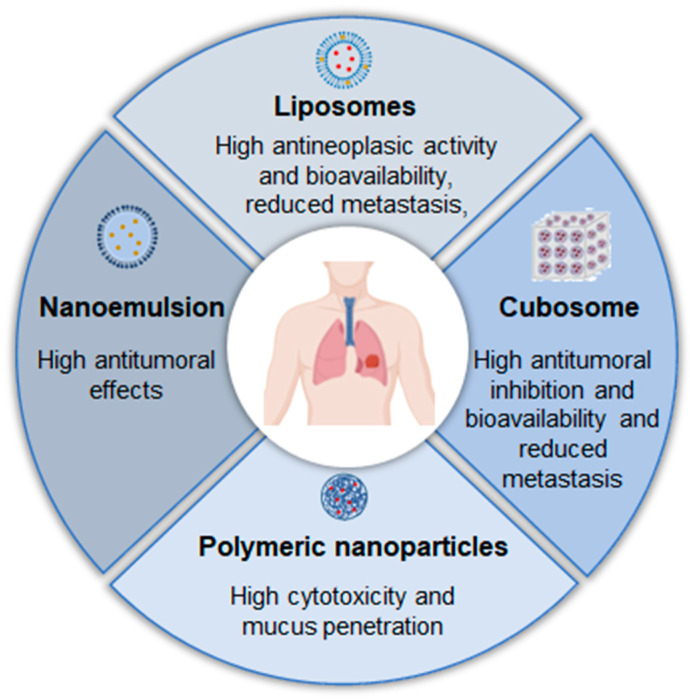
Examples of nanostructured systems already studied and their advantages for local treatment of lung cancer.

In summary, nanostructured formulations can also be a promising option for the local treatment of lung cancer, offering several advantages in efficacy (Figure 6).

### 4.5. Melanoma

Melanoma is a type of cancer that arises from the malignant transformation of melanocytes. It is classified into three subtypes: cutaneous melanoma, which arises from melanocytes in the epidermis; mucosal melanoma, which arises from melanocytes residing in the mucous membranes; and uveal melanoma, which arises from melanocytes residing in the ocular stroma [87]. The most common of these is cutaneous melanoma, which is the 17th most common cancer worldwide, with 331,647 new cases, and the 22nd leading cause of death among all cancers, resulting in 58,645 deaths in 2022 [3]. A variety of nanostructured nanoformulations have been developed for the local treatment of melanoma via intratumoural and topical routes, as outlined in Table 5.

Kim et al., 2020 [90] developed nanoparticles with polymerized β-cyclodextrin (pCD) and polymerized paclitaxel (pPTX), followed by the S-nitrosylation of thiol groups in pCD-pSH for locoregional cutaneous melanoma chemoimmunotherapy. The resultant pPTX/pCD-pSNO nanoparticles exhibited significantly enhanced cytotoxicity and immunogenic cell death in vitro. In vivo, intratumoural administration activates and expands dendritic cells systemically; however, it also leads to an increase in myeloid-derived suppressor cells and inhibits CD8+ T cell expansion. When paired with an antibody that targets cytotoxic T lymphocyte antigen-4, which diminishes its suppressive signalling on T cells, the intratumoural pPTX/pCD-pSNO treatment demonstrates robust anticancer activity, significantly extending survival in animal models.

In addition to intratumoural injection, alternative local administration mechanisms may be employed in cutaneous melanoma, including the cryomicroneedle system developed by Shi et al. (2024) [88]. Gold nanoparticles were conjugated to the bacteria *Rhodospirillum rubrum* with the aim of achieving photochemical transformation. The nanogold-engineered system (R.r-Au) was administered transdermally to the skin tumour of mice via a cryomicroneedle patch following laser application. Following irradiation, R.r-Au facilitated enhanced electron transfer by activating Au nanoparticles within the photosynthetic system of *R. rubrum*. This process increased lactate consumption and hydrogen production, resulting in heightened tumour immune activation and a subsequent reduction in tumour volume, weight, and size. The developed system was considered a minimally invasive in situ delivery approach for living bacterial drugs with biosafety, since no histopathological lesions were observed in other organs and the biochemical indicators did not have any significant differences.

Additionally, nanocarriers have been developed for the topical treatment of melanoma. The chemotherapeutic agent 5-fluorouracil and the photosensitizer imperatorin were co-encapsulated in transferosomes, a type of liposome characterized by highly elastic vesicles, embedded in Carbopol gel [91]. The nanosystem demonstrated enhanced skin permeability and selectivity for the active compounds, accompanied by a reduction in tumour volume and weight in mice exposed to UVA laser sources.

Topical application has also been employed for the ocular administration of nanoparticles for the treatment of uveal melanoma. For example, uncoated and polyethylene glycol-coated nanostructured lipid carriers were developed for the ophthalmic delivery of (S)-(–)-MRJF22, a novel prodrug with potential utility in the treatment of uveal melanoma [89]. These carriers were deemed appropriate for ocular application due to their small particle size, mucoadhesive properties, and low irritant potential. In vitro and in vivo studies demonstrated that the nanoparticles exhibited high selective cytotoxicity and antiangiogenic and anti-inflammatory effects in comparison to the free prodrug. Biodistribution imaging in rabbits indicated that the nanoparticles could reach the eye’s posterior segment, thus offering a promising strategy for treating choroidal uveal melanoma.

**Figure 7:**
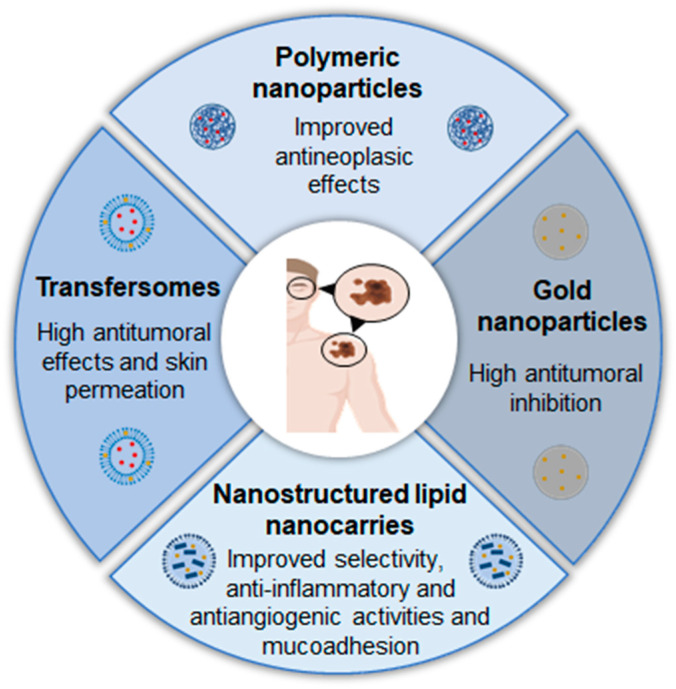
Examples of nanostructured systems already studied and their advantages for the local treatment of cutaneous and uveal melanoma.

In summary, nanostructured formulations is being studied as a promising drug delivery system for intratumoural or topical administration, offering several advantages in efficacy (Figure 7).

### 4.6. Oral Cancer

Oral cancers can develop on the tongue, the tissue lining the mouth and gums, under the tongue, the base of the tongue, and the throat area at the back of the mouth. While early diagnosis is relatively straightforward, many cases still present at an advanced stage [92]. The primary causes are excessive alcohol consumption and tobacco use [93]. However, additional risk factors, such as prolonged sun exposure [94] and HPV infection [95], have also been linked to its development.

The primary treatment for oral cancer is surgery. However, combining surgery with chemotherapy and radiotherapy can lead to better outcomes, particularly in advanced cases [92]. To enhance treatment efficacy and minimize side effects, nanotechnology-based drug delivery systems offer a promising approach [96]. In this context, we discuss several examples of these nanocompositions, with a focus on their potential for the local treatment of oral cancer (Table 6).

Pornpitchanarong et al. (2020) [99] developed catechol-modified chitosan/hyaluronic acid nanoparticles as a novel delivery system for doxorubicin to target oral cancer cells. These nanoparticles were engineered with catechol groups to enhance their mucoadhesive properties, allowing them to adhere effectively to the oral mucosa. The mucoadhesive design increased the retention time of the nanoparticles at the site of action and provided sustained release of doxorubicin. A flow-through mucoadhesion study using porcine mucosa demonstrated excellent adhesion, validating the potential of the developed formulation for localized treatment. In vitro, cellular uptake of the doxorubicin-loaded nanoparticles was significantly higher than that of free doxorubicin, suggesting improved drug delivery to oral cancer cells.

Lin et al. (2020) [97] developed mitochondria-targeting nanofibers for enhanced photodynamic cancer therapy. These nanofibers were formed via the self-assembly of amphiphilic small molecule building blocks composed of pheophorbide and quinolinium conjugates. Pheophorbide, a hydrophobic photosensitizer, facilitated ROS production under light irradiation, while the hydrophilic quinolinium moiety ensured selective targeting to mitochondria. The nanofibers demonstrated significantly higher cytotoxicity against oral cancer cells compared to free pheophorbide, an effect amplified by light treatment. Importantly, non-tumoural cells exhibited greater tolerance to nanofibers, with or without light irradiation, indicating selectivity toward cancer cells. Enhanced cellular uptake of nanofibers was observed, driven by their homogeneous nanosize and positively charged surface, resulting in pronounced mitochondrial accumulation. Under light exposure, nanofibers produced ROS levels approximately 110-fold higher than free pheophorbide at the same concentration, driving effective cancer cell death. In vivo, intratumoural injection of nanofibers in OSC-3 tumour-bearing mice resulted in prolonged fluorescence signals at the tumour site, lasting nearly a week longer than the free drug. In both subcutaneous and orthotopic oral cancer models, light-activated nanofibers achieved complete tumour growth inhibition.

A targeted photothermal therapy platform using gold nanorods functionalized with Shiga toxin-B was developed by Navarro-Palomares et al. (2022) [98]. This protein, derived from *Shigella dysenteriae* and certain *Escherichia coli* strains, binds specifically to globotriaosylceramide receptors, which are overexpressed on the membranes of many cancer cells [100]. The targeted nanorods efficiently interacted with globotriaosylceramide receptors, enabling precise localization to oral cancer cells. Upon laser illumination, the gold nanorods generated hyperthermia, selectively killing the targeted cancer cells. In vitro studies demonstrated that Shiga toxin-B-functionalized nanorods induced significantly higher cytotoxicity under light stimulation compared to non-functionalized nanorods or those used without light treatment. In vivo, in a murine oral cancer model, the combined treatment of Shiga toxin-B nanorods and light irradiation effectively eliminated cancer cells. Similar results were observed in 3D human cell cultures derived from clinical biopsy specimens of oral carcinoma, thereby confirming the effectiveness of the nanocomposition in a clinically relevant context.

Goldberg et al. (2022) [34] explored a self-adhesive transmucosal system for the targeted delivery of cisplatin to treat oral cancer. This system, known as PRV111, features a thin, two-layer polymeric patch designed to overcome the challenges of drug washout and systemic toxicity associated with cisplatin administration. PRV111 consists of a chitosan matrix layer embedded with cisplatin-loaded chitosan particles and an impermeable ethyl cellulose backing, which ensures localized drug delivery while preventing particle washout from saliva. Additionally, the patch incorporates a permeation enhancer that temporarily opens tight junctions between cells, facilitating optimal drug penetration and absorption. Preclinical studies demonstrated substantial tumour size reduction observed after just three treatments, with complete tumour regression in most animals by day 17. Tumour tissues from PRV111-treated animals showed significantly higher cisplatin levels compared to the free cisplatin treatment, while other tissues displayed negligible cisplatin levels, indicating minimal systemic exposure. Also, animals in the PRV111 group remained tumour progression-free for at least 10 days post-treatment. Encouraged by these results, a phase I/II clinical trial evaluated the safety and efficacy of PRV111 for oral cancer. The treatment led to a rapid and significant decrease in tumour size without serious adverse events or systemic toxicities. Furthermore, no locoregional recurrences were observed within six months of treatment.

**Figure 8:**
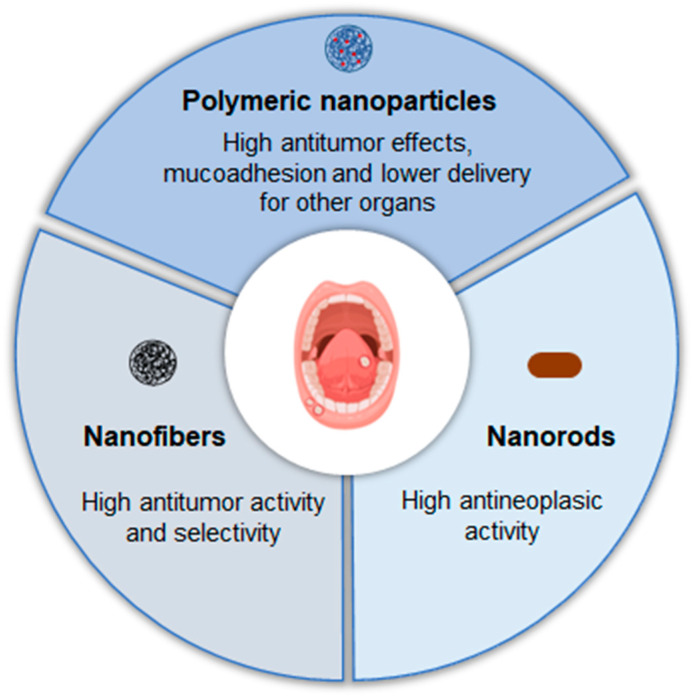
Examples of nanostructured systems already studied and their advantages for the local treatment of oral cancer.

In summary, nanostructured formulations is currently under investigation with beneficious results for the local treatment of cutaneous and uveal melanoma via intratumoural or topical administration, offering several advantages in efficacy (Figure 8).

## 5. Current Challenges

Despite the evidence for efficacy, nanotechnology presents certain challenges, including the difficulties in issues related to the production process and the lack of regulation and standards, which make the transition from the laboratory to the market difficult [101,102].

### 5.1. Challenges in the Production Process

During the preclinical phase, a series of assays are conducted to optimize the production of nanostructures, with the objective of meeting specific size, PDI, drug content, encapsulation efficiency, drug release, and stability criteria [103,104,105]. However, the majority of studies neglects to address the scale-up of the process, and consequently, challenges such as the difficulties involved in large-scale synthesis, the generation of waste, and elevated production costs impede the manufacturing of nanoformulations for clinical studies and market deployment [101,102].

During the scale-up of a laboratory method, the desired features of nanoparticles are often compromised [101]. The parameters used in the small-scale laboratory synthesis must be re-optimized due to differences in fabrication techniques for clinical production [106]. In addition, the production method must be appropriately selected since the preclinical phase by considering the scale-up process, thereby enabling the develop a variety of nanomedicines for diverse applications [101].

In relation to the issue of synthesis waste, the green synthesis of nanoparticles, which employs plant extracts in place of industrial chemical agents, has been developed as a means of addressing environmental pollution, high energy consumption, and potential health concerns [107]. The green synthesis of nanoparticles of gold, silver, palladium, copper, and iron and its oxide has been the subject of recent research, but the production process is constrained by the complexity of geographical and seasonal distributions of plants, as well as issues with low purity and poor yield [107]. A meticulous examination of the materials, solvents, and nanoparticle fabrication methods is imperative to circumvent the myriad of challenges associated with nanoparticle production for clinical application [101].

### 5.2. Regulatory and Clinical Issues

Even when optimal production can be achieved at a scale with acceptable yield and minimal waste, there are no global regulatory standards for clinical trials of nanotherapeutic drugs. Market translation is limited by the absence of regulations and standards governing manufacturing procedures, quality control, safety and efficacy assessment, and translation [102]. For example, of all the studies presented in this review, only one included a clinical trial: the self-adhesive transmucosal system for targeted delivery of cisplatin to treat oral cancer [34].

Regulatory agencies such as the Food and Drug Administration of United State of America (USFDA), European Medicines Agency (EMA), Medicines and Healthcare Products Regulatory Authority of United Kingdom (MHRA), and Therapeutic Goods Administration of Australia (TGA) are in continuous efforts to achieve a regulatory framework on nano-specific products; however, no global regulatory standards exist [102,108]. Unfortunately, the development of nanomedicines moves quicker than the regulatory landscape, and frameworks and a worldwide cooperation in the regulatory area are necessary to address concerns regarding their associated quality, safety, efficacy, and toxicity issues [108].

Despite the challenges associated with translating nanomedicines into clinical trials, some have already been clinically approved by regulatory agencies. Examples include liposomes (Doxil^®^, Myocet^®^, DaunoXome^®^, and Onivyde^®^) and polymer micelles (Genexol^®^, Nanoxel^®^, and Apealea^®^) [109]. These pharmaceuticals have been developed to treat various cancers, offering prolonged circulation time and reduced side effects [109].

## 6. Perspectives and Trend of Local Nanosystems in Cancer

Notwithstanding the challenges previously mentioned, research involving nanoformulations for the local treatment of cancer has made significant advances. In addition to the efficacy of nanocarriers in delivering chemotherapy, gene therapy, and immunotherapy locally to the tumour, there is also a current trend in research towards the development of these nanostructures as theranostic agents or associate the treatment with irradiation methods.

### 6.1. Theranostics

The term ‘theranostic’ represents a revolutionary concept in cancer treatment whereby biocompatible nanocarriers co-deliver imaging and pharmaceutical agents, thereby combining therapeutic and diagnostic functions in a single system. This approach has the potential to enhance the effectiveness of treatment, overcome tumour resistance, and modulate the tumour microenvironment [110].

For example, covalent polymer nanoparticles, as a novel platform for radiolabeling with Lutetium-177 (^177^Lu), were developed for breast cancer [111]. ^177^Lu is a radionuclide with a half-life of 6.64 days, emitting γ-rays suitable for nuclear bioimaging and β-particles ideal for tumour endoradiotherapy [112]. In vitro, the nanoformulation exhibited enhanced cellular uptake by 4T1 breast cancer cells and induced higher cytotoxicity rates compared to free ^177^Lu. In vivo, intratumoural injections of the nanoparticles significantly reduced tumour size, with no recurrence observed within a 90-day follow-up period. Survival rates were markedly extended, and the nanoparticles demonstrated prolonged tumour retention for up to 48 h, with a minimal off-target distribution.

Other examples of theranostic nanosystems are the zinc gallate (ZnGa_2_O_4_)-based luminescence nanoparticles functionalized with poly(vicinal diol) that were developed for an innovative theranostic approach for melanoma on the basis of the boron neutron capture therapy. In vitro assays showed that the formulation was internalized substantially by WEHI-164 cancer cells that have potent cytotoxicity. Furthermore, in vivo studies in mice with intratumoural administration evidenced the accumulation of this formulation at the acidic tumour site with tumour volume reduction (75−80%) after treatment with nanoformulation and neutron irradiation. The use of formulation also demonstrated the safety of the treatment since the histopathological studies indicated no signs of any damage to the vital organs [113].

### 6.2. Nanoformulations Associated with Irradiation Methods

The combination of nanoformulations with conventional physical methods, such as radiotherapy, is a novel approach to local cancer treatment. This integration has the potential to yield synergistic abscopal effects, which could contribute to sustained therapeutic responses [13]. The study performed by Sharma et al., 2022 [113], cited above and with other examples mentioned in this review, demonstrated the potential of this approach to enhance the efficacy of the local treatment.

Another example is a transdermal drug delivery cream containing a liposome-encapsulated Ru(II) complex developed for the treatment of skin cancer. The association of the cream of irradiation with an 808 nm laser ensures an adequate absorption and accumulation of active compounds in the tumours, thereby allowing similarly effective therapeutic outcomes in comparison to intravenous injection [114].

## 7. Conclusions

The employment of nanostructured formulations holds considerable promise as a localized cancer treatment modality, as evidenced by the examples presented. These formulations have the potential to facilitate drug penetration and enhance drug concentration within tumours, thereby minimizing off-target effects and optimizing treatment efficacy while reducing adverse effects. Notwithstanding the challenges associated with physiology, the production process, and regulatory issues, the benefits of cancer treatment with the local administration of nanocarriers are evident. Consequently, it is strongly recommended that studies continue to be conducted to identify solutions to the challenges and achieve effective treatment options that will either cure or considerably prolong the quality of life of cancer patients.

## Figures and Tables

**Table 1:** Nanostructured formulations for the local treatment of bladder cancer.

Nanosystem	Particle Size and Polydispersity Index (PDI)	Active Compound	Model Assay	Therapy Method (In Vivo)	Results	Reference
Polymeric nanoparticles	83.4 nm PDI 0.183	Gambogic acid 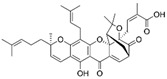	In vitro (MB49 cells) and in vivo (C57BL/6 J mice)	Intravesical instillation	↑ Cytotoxicity ↑ Selectivity ↑ Mucoadhesiveness ↑ Bladder penetration ↑ Anti-tumour efficacy	[39]
Polypeptide nanogel		10-hydroxycamptothecin 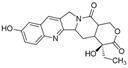	In vitro (5637 cells) and in vivo (Sprague Dawley rats and C57Bl/6 mice)	Intravesical instillation	↑ Cellular uptake ↑ Cytotoxicity ↑ Mucoadhesiveness ↑ Bladder penetration ↓ Delivery for other organs ↑ Anti-tumour effects ↓ Toxicity ↑ survival	[40]
Silica nanoparticles	124.5 PDI 0.48	Doxorubicin 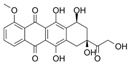	In vitro (porcine bladder and UM-UC-3 cells)	_	↑ Mucoadhesiveness	[41]

↑: increase, ↓: decrease.

**Table 2:** Nanostructured formulations for the local treatment of breast cancer.

Nanosystem	Particle Size and Polydispersity Index (PDI)	Active Compound	Model Assay	Therapy Method (In Vivo)	Results	Reference
Liposomes in hydrogel	135.0 PDI 0.11	Gambogic acid 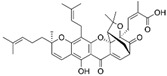	In vivo (BALB/c mice)	Intratumor injection	↑ anti-tumour efficacy	[45]
Magnetic nanoparticles	36.0 nm	Doxorubicin 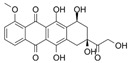	In vitro (4T1 and MCF-7 cells) and in vivo (BALB/c mice)	Intratumor injection	↑ cellular uptake ↑ anti-tumour efficacy	[46]
Nanosuspension (NS) and nanoparticle (NP)	NS: 207.8 nm, PDI 0.12 NP: 189.1 nm, PDI 0.17	Ciclopirox 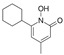	In vitro (13762 Mat B III, MCF-7, and MDA-MB-231 cells) and in vivo (F344 rats)	Intraductal administration	↑ mammary persistence, ↑ anti-tumour effects	[47]
Polymeric nanoparticles	Not informed	Cupric ion Cu^2+^	In vitro (MCF-7, A549 and MDA-MB-231 cells) and in vivo (mice)	Intratumor injection	↑ selectivity ↑ anti-tumour efficacy	[48]

↑: increase, ↓: decrease.

**Table 3:** Nanostructured formulations for the local treatment of colorectal cancer.

Nanosystem	Particle Size and Polydispersity Index (PDI)	Active Compound	Model Assay	Therapy Method (In Vivo)	Results	Reference
Carbon-based nanoparticle	129.2 nm PDI 0.14	Folic acid-modified 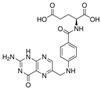	in vitro (HCT-116; L02; NIH3T3; NCM460) and in vivo (C57BL/6 mice)	Oral	↑ Targeting ↑Accumulation in colon ↑ Residence time in colon ↑ Cytotoxicity ↓ Immune inflammatory response ↑ Anti-tumour effects	[60]
Liposome	221.3 nm	IVT IL-15 mRNA	in vitro (293T; 3T3; C26) and in vivo (female BALB/c mice)	Intratumor injection	↑ Cytotoxicity ↑ Anti-tumour effects ↓ Angiogenesis ↓ Metastasis	[61]
Nanocrystal	225.7 nm PDI 0.23	Bufadienolides 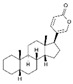	in vitro (CT-26; HT-29) and in vivo (BALB/c)	Oral	↑ Uptake ↑ Cytotoxicity ↑ Targeted intestinal sites ↑ Retention time ↑ Oral bioavailability ↑ Anti-tumour effects	[62]
Nanodiamonds	221.4 nm PDI 0.20	Doxorubicin hydrochloride 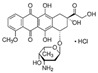	in vitro (CT-26) and in vivo (BALB/c mice)	Oral	↑ Photothermal conversion ↑ Cytotoxicity ↑ Accumulation in colon ↑ Anti-tumour effects	[63]
Nanohydrogel	174.8 nm PDI 0.06	Methotrexate and chloroquine 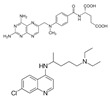	in vitro (SW480; HFF2) and in vivo (Swiss albino mice)	Oral	↑ Cytotoxicity	[64]
Nanosuspension	19.6 nm PDI 0.06	5-fluorouracil 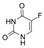	in vitro (HCT-116)	Oral	↑ Cytotoxicity	[65]
Pectin nanoparticle	12.0 nm	Chlorogenic acid 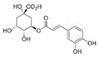	in vitro (HCT-116)	Oral	↑ Cytotoxicity	[66]
Peptosome	53.0 nm	Anti-miR-31 oligonucleotide and Curcumin 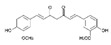	in vitro (HCT116; LoVo; HT29-MTX) and in vivo (male C57BL/6 mice)	Rectal and oral	↑ Mucoadhesion ↑ Drug retention ↑ Local bioavailability ↑ Uptake ↑ Cytotoxicity ↑ Anti-tumour effects	[67]
Polymeric nanoparticle	180 nm	IR780 and 5-ALA 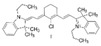	in vitro (Caco-2; CT-26) and in vivo (male BALB/c mice)	Oral	↑ Cytotoxicity ↑ Biodistribution ↑ Anti-tumour effects	[68]
Polymeric nanoparticle	60.0 nm	Doxorubicin 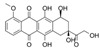	in vitro (HT29; CHO)	--	↑ Cytotoxicity	[69]
Polymeric nanoparticle in hydrogel	233.11 nm PDI 0.23	Diferourylmethane 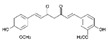	in vitro (HeLa; A549) in vivo (Rabbit’s)	Oral	↑ Uptake ↑ Cytotoxicity ↑ Peak maximum concentration ↑ Residence time	[70]
Solid lipid nanoparticles in microgel	116.0 nm	Cisplatin/superparamagnetic iron oxide 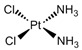	in vitro (CT-26) and in vivo (male BALB/c mice)	Oral	↑ Cytotoxicity ↑ Accumulation in colon ↓ Tumour growth ↓ Metastasis	[71]

↑: increase, ↓: decrease.

**Table 4:** Nanostructured formulations for the local treatment of lung cancer.

Nanosystem	Particle Size and Polydispersity Index (PDI)	Active Compound	Model Assay	Therapy Method (In Vivo)	Results	Reference
Cubosomes	150.2 nm PDI 0.24	Bedaquilina 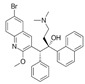	In vitro (A549)	_	↑Bioavailability ↑ Cytotoxicity ↓ Metastasis ↑ Anti-tumour effects	[77]
Liposome	211.8 nm PDI 0.30	Pirfenidone 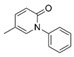	In vitro (NSCLC A549; H460; H4006 ) and ex vivo	_	↑ Cytotoxicity ↑ Anti-tumour effects ↓ Metastasis	[78]
Liposome	64.3 nm PDI 0.35	Paclitaxel 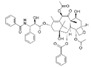	In vitro (A549) and in vivo (Male Sprague–Dawley rats)	Intratracheally (i.t.) sprayed	↑Bioavailability ↑ Immune response	[79]
Liposome	119.0 nm PDI 0.20	Indomethacin 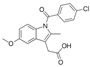	In vitro (NSCLC A549; H1299; H460)	_	↑Bioavailability ↑ Cytotoxicity ↓Tumour progression	[80]
Liposome	100.9 nm PDI 0.20	Osimertinib 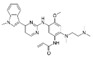	In vitro (H-1975, H1975)	_	↑Bioavailability ↑ Cytotoxicity ↑ Anti-tumour effects	[81]
Nanoemulsion	122.2 nm PDI 0.36	Tyrosine kinase inhibitor	in vitro (NSCLC A549) and ex vivo	_	↑ Cytotoxicity ↑ Anti-tumour effects	[82]
Polymeric nanoparticle	119.0 nm	K4 monomer	In vitro (Lewis lung carcinoma) and in vivo (C57BL/6N mice)	Intratracheal instillation	↑ Cytotoxicity ↑Mucus penetration	[83]
Polymeric nanoparticle	187.2 nm PDI 0.28	Gefitinib 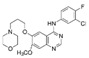	In vitro (A549)	_	↑ Cytotoxicity ↑ Cellular internalization	[84]
Polymeric nanoparticle	191.6 nm PDI 0.04	Niclosamida 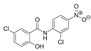	In vitro ( A549) and in vivo (Balb/c mice)	Inhalation	↑ Cytotoxicity ↑Cellular internalization	[85]

↑: increase, ↓: decrease.

**Table 5:** Nanostructured formulations for the local treatment of melanoma cancer.

Nanosystem	Particle Size and Polydispersity Index (PDI)	Active Compound	Model Assay	Therapy Method (In Vivo)	Results	Reference
Gold nanoparticles	15 nm	*Rhodospirillum rubrum*	In vivo (Balb/c mice)	**Transdermal**	↑ Tumour inhibition	[88]
Nanostructured lipid carriers	160.0 nm PDI 0.25	(S)-(–)-MRJF22 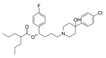	In vitro (92–1 and HCE-2 cells and chorioallantoic membrane), in vivo (New Zealand albino rabbits)	Ocular	↑Selectivity ↑ Anti-inflammatory activity ↑ Antiangiogenic activity ↑ Mucoadhesion	[89]
Polymeric nanoparticles	72.4 nm	Paclitaxel and nitric oxide 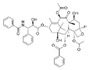	In vitro (B16F10,), in vivo (C57BL/6 mouse)	Dorsal injection	↑Cytotoxicity ↑ Immunogenic cell death ↑ Tumour suppression ↑ Animal survival.	[90]
Transfersomes	50.0 nm PDI 0.20	5-fluorouracil and imperatorin 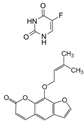	In vitro (A375 and HaCaT), in vivo (BALB/c-Nu mice)	Subcutaneous injection	↑ Skin permeability ↑ Selectivity ↓ Volume and weight tumour	[91]

↑: increase, ↓: decrease.

**Table 6:** Nanostructured formulations for the local treatment of oral cancer.

Nanosystem	Particle Size and Polydispersity Index (PDI)	Active Compound	Model Assay	Therapy Method (In Vivo)	Results	Reference
Nanofibers	14.9 nm	Pheophorbide 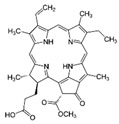	In vitro (OSC-3 cells) and in vivo (athymic nude mice)	Intratumor injection	↑ Cytotoxicity ↑ Selectivity ↑ Cellular uptake ↑ Mitochondria accumulation ↑ ROS production ↑ Anti-tumour effects	[97]
Nanoparticles	Not informed	Cisplatin 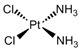	In vivo (golden Syrian hamster) and clinical trial	Intratumor injection and local application	↑ Anti-tumour effects ↓ Delivery for other organs Safe and effective in humans	[34]
Nanorods	Not informed	-	In vitro (Detroit 562 cells and biopsy samples) and in vivo (C57BL/6 mice)	Oral	↑ Cellular uptake ↑ Cytotoxicity ↑ Anti-tumour efficacy	[98]
Polymeric nanoparticles	239.4 nm PDI 0.30	Doxorubicin 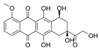	Ex vivo (porcine oral mucosa) and in vitro (HN22 cells)	_	↑ Mucoadhesiveness ↑ Cellular uptake	[99]

↑: increase, ↓: decrease.

## Data Availability

Data are contained within the article.
